# Effect of carboxymethyl cellulose and gibberellic acid-enriched biochar on osmotic stress tolerance in cotton

**DOI:** 10.1186/s12870-024-04792-4

**Published:** 2024-02-26

**Authors:** Lisheng Qian, Shoucheng Huang, Zhihua Song, Shah Fahad, Khadim Dawar, Subhan Danish, Hina Saif, Khurram Shahzad, Mohammad Javed Ansari, Saleh H. Salmen

**Affiliations:** 1 College of Life and Health Science, Anhui Science and Technology University, Fengyang, 233100 China; 2 College of Food Science, Anhui Science and Technology University, Fengyang, 233100 China; 3 Department of Agronomy, Abdul Wali Khan University Mardan, Mardan, 23200, Khyber Pakhtunkhwa Pakistan; 4 Department of Soil and Environmental Science, the University of Agriculture Peshawar, Peshawar, Pakistan; 5 Department of Soil Science, Faculty of Agricultural Sciences and Technology, Bahauddin Zakariya University, Multan, Punjab Pakistan; 6 Department of Environmental Sciences, Woman University Multan, Multan, Punjab Pakistan; 7 Department of Soil Science, University College of Dera Murad Jamali, LUAWMS, Dera Murad Jamali, Balochistan Pakistan; 8 Department of Botany, Hindu College Moradabad (MJP Rohilkhand University Bareilly), Moradabad, 244001 India; 9 Department of Botany and Microbiology, College of Science, King Saud University, PO Box -2455, Riyadh, 11461 Saudi Arabia

**Keywords:** Carboxymethyl cellulose, Osmotic stress, Biochar, Chlorophyll content, Reactive oxygen species

## Abstract

The deleterious impact of osmotic stress, induced by water deficit in arid and semi-arid regions, poses a formidable challenge to cotton production. To protect cotton farming in dry areas, it’s crucial to create strong plans to increase soil water and reduce stress on plants. The carboxymethyl cellulose (CMC), gibberellic acid (GA_3_) and biochar (BC) are individually found effective in mitigating osmotic stress. However, combine effect of CMC and GA_3_ with biochar on drought mitigation is still not studied in depth. The present study was carried out using a combination of GA_3_ and CMC with BC as amendments on cotton plants subjected to osmotic stress levels of 70 (70 OS) and 40 (40 OS). There were five treatment groups, namely: control (0% CMC-BC and 0% GA_3_-BC), 0.4%CMC-BC, 0.4%GA_3_-BC, 0.8%CMC-BC, and 0.8%GA_3_-BC. Each treatment was replicated five times with a completely randomized design (CRD). The results revealed that 0.8 GA_3_-BC led to increase in cotton shoot fresh weight (99.95%), shoot dry weight (95.70%), root fresh weight (73.13%), and root dry weight (95.74%) compared to the control group under osmotic stress. There was a significant enhancement in cotton chlorophyll a (23.77%), chlorophyll b (70.44%), and total chlorophyll (35.44%), the photosynthetic rate (90.77%), transpiration rate (174.44%), and internal CO_2_ concentration (57.99%) compared to the control group under the 40 OS stress. Thus 0.8GA_3_-BC can be potential amendment for reducing osmotic stress in cotton cultivation, enhancing agricultural resilience and productivity.

## Introduction

The deleterious impact of osmotic stress, induced by water deficit in arid and semi-arid regions, poses a formidable challenge to cotton production [1]. Drought conditions lead to a lessened water potential in plant cells, impeding the uptake of water through roots and causing a disruption in the delicate balance of cellular osmotic equilibrium [2]. This osmotic stress, exacerbated by the scarcity of water in the soil, emerges as a critical bottleneck in cotton cultivation, severely compromising productivity [3]. Recognizing the imperative to safeguard cotton production in arid and semi-arid areas, there arises a critical need to formulate and implement robust strategies aimed at augmenting soil water content and mitigating osmotic stress. Cellulose is the most abundant organic material in nature, completely insoluble in water, carboxymethyl enhances its solubility. CMC is a natural polymeric coating material that exhibits a multitude of beneficial properties and is suitable for water retention and soil conditioning under a stress environment [4]. It enhances soil water holding capacity and improves plant water utilization, negative charges on the carboxymethyl group interact with the cations of the soil solution and inhibit the stress condition [5].

Gibberellic acid (GA_3_), produced from *Gibberella fujikuroi*, is a multifunctioning signaling, essential plant hormone and growth regulator that has been reported to stimulate plants’ biochemical as well as physiological processes especially under stressed environment [6–8]. GA_3_ contributes to seed sprouting, stem elongation, floral instigation, signaling, cell expansion, fruit development, stem elongation, net photosynthetic rate, carbohydrate metabolism, antioxidant system, and water uptake regulation [9–11]. Because of the multifunctional of GA_3_, it can be efficiently used against osmotic stress.

Biochar a carbon-rich material, relative form of charcoal produce form wide range of biomass includes woody material, livestock material, crop straw and organic waste. It is a promising ameliorant that can improve the crop growth by modulating soil conditions due to its unique characteristics such as large surface area (SA) abundant oxygen (O)-containing functional groups, rich pore structure, and high cation exchange capacity (CEC) [12, 13].

The combined effect of CMC and GA_3_ enriched biochar on drought mitigation in cotton production is still not well studied. The current study was to evaluate the combined effect of CMC and GA_3_ enriched biochar on drought mitigation in cotton. The study aims to understand the mechanisms by which biochar facilitates CMC and GA_3_ and scrutinize the effect of CMC and GA_3_ along with biochar on cotton production under osmotic stress. It is hypothesized that the use of CMC and GA_3_ along with biochar might regulate the nutrient balance, antioxidant activity, photosynthesis, and stomata conductance in cotton plants and mediate the toxic effect of Osmotic stress.

## Material and methods

### Experimental site

In the experimental site of Research Solution, a pot trial was conducted. Soil samples were accurately acquired from the Chenab River’s bank, situated in Multan, Punjab, Pakistan, (30°19′20.8"N 71°24′50.4"E).

#### Biochar preparation and characterization

The preparation of biochar involved the conversion of cotton sticks (waste material), through pyrolysis at a temperature of 440 °C. Following cooling, the biochar was finely ground to a size of 2 mm and stored for future applications. The composition of the biochar was determined using gravimetric analysis, following the methodology outlined by [14]. To assess the pH, as described by Page et al. in 1983, and electrical conductivity (EC) following the method by [15], a 1:10 mixture of biochar and distilled water was prepared, and portions of this mixture were subjected to analysis. The determination of nitrogen (N) concentration in the biochar was performed by digesting and distilling biochar samples, employing the Kjeldahl distillation method as detailed by [16]. For the assessment of phosphorus (P) and potassium (K^+^) concentration in the biochar, a mixture of HNO_3_-HClO_4_ was utilized as outlined by [17]. Subsequently, the phosphorus (P) concentration was analyzed using a spectrophotometer, employing the ammonium vanadate-ammonium molybdate yellow color technique, while the potassium (K^+^) concentration was determined using a flame photometer according to [18].

### CMC-BC and GA_3_-BC

The specific CMC product used was identified by its Product Number 21904 and Batch Number BCCG3327. This CMC product was branded as SIGMA and had a CAS Number of 9004–32-4. The CMC’s viscosity was measured at 3100 mPa.s, which falls within the acceptable range of 1500 to 4500 mPa.s. To create the CMC-enriched biochar, 30 mg of CMC was mixed with the biochar by adding deionized water. After complete mixing the material was incubated for 72 h at 25 ± 2 °C. Finally, oven drying was done to achieve a dried powder.

For making GA_3_ enriched biochar, 10 g of 10% GA_3_ (CAS 77-06-5) was mixed in the 10 kg of biochar powder. The mixing was done manually before biochar application in soil to avoid any losses of GA_3_.

### Pot experiment

The cotton seeds (NIAB-878) were procured from certified seed dealer of government of Punjab, Multan, Pakistan. Damaged or weak seeds were intentionally excluded manually. In preparation for planting, the chosen seeds underwent a surface-sterilization procedure. This involved subjecting the seeds to a solution of 5% sodium hypochlorite, followed by three subsequent rinses with 95% ethanol. To eliminate any residual traces of the sterilizing agents, the seeds were further subjected to three thorough washes using deionized water that had been sterilized [19]. The air temperature ranged from 17 °C to 32 °C, with humidity varying between 40 and 62% in the greenhouse. Soil from the station field, a type called clay loam, was collected through a 2-mm sieve and placed into plastic pots in layers. Each pot contained 5 kg of dried soil. The soil was packed at bulk density of 1.2 g/cm^3^. Each pot had a height of 40 cm, width of 31 cm and volume of 9.6 L. The 10 seeds were planted in each pot. After 15 days of germination, only 5 seeds were maintained in each pot. The treatments (Control, 0.4% CMC-BC, 0.4% GA 3-BC, 0.8% CMC-BC, 0.8% GA 3-BC) were mixed at the time of pot filling. To induce no osmotic stress (OS) 70% and osmotic stress 40% field capacity were maintained, throughout the trial [20]. This was achieved using a moisture meter (YIERYI 4 in 1; Shenzhen, Guangdong Province, China) for precise management of irrigation. After a 50-day growth period from the time of sowing, the plants were harvested to collect data.

### Data collection

Subsequently, the fresh weights of both shoots and roots were measured immediately post-harvest. To determine the dry weights of the shoots and roots, samples were subjected to oven drying at 65 °C for 72 h until a constant weight was attained.

### Chlorophyll contents and carotenoids

To quantify the chlorophyll a, chlorophyll b, and total chlorophyll contents in freshly harvested cotton leaves, we followed a procedure inspired by Arnon method [21]. The extraction process involved the use of an 80% acetone solution. The absorbance readings were obtained at distinct wavelengths: 663 nm for chlorophyll a, 645 nm for chlorophyll b and 470 nm for carotenoids.

$$\text{Chlorophyll}\;\text{a}\;\left(\frac{\mathrm{mg}}{\text{g}}\right)=\frac{\left(12.7\;\times\;\mathrm{A}663\right)\;-\;\left(2.69\;\times\;\mathrm{A}645\right)\;\times\;\mathrm{V}}{1000\;\times\;\text{W}}$$

$$\text{Chlorophyll}\;\text{b}\left(\frac{\mathrm{mg}}{\text{g}}\right)\;=\;\frac{(22.9\times {\mathrm{A}}645)\;-\;(4.68\;\times\;{\mathrm{A663}})\;\times {\mathrm{V}}}{1000\;\times\;{\text{W}}}$$

$$\mathrm{Total\ Chlorophyll }\left(\frac{{\mathrm{mg}}}{{\text{g}}}\right)=\frac{20.2\left({\mathrm{A}}645\right)+8.02\left({\mathrm{A}}663\right)\times {\mathrm{V}}}{1000 \times {\text{W}}}$$

$$\mathrm{Carotenoids }\left(\frac{\mathrm{mg}}{\text{g}}\right)={\text{OD}}480+0.114 \left({\text{OD}}663\right)-0.638 ({\text{OD}}645)$$

### Gas exchange attributes

The net photosynthetic rate, transpiration rate, and stomatal conductance were assessed using an Infra-Red Gas Analyzer (the CI-340 Photosynthesis system by CID, Inc. USA). These measurements were taken on a sunlit day from 10:30 to 11:30 AM when light intensity was at a level conducive for photosynthesis [22].

### Antioxidants

The activity of superoxide dismutase (SOD), we measured the inhibition of nitro blue tetrazolium (NBT) reduction in the presence of riboflavin. The reaction mixture, comprising enzyme extract, NBT, riboflavin, and phosphate buffer, was subjected to illumination, and changes in absorbance at 560 nm were continuously recorded [23]. To evaluate peroxidase (POD) activity, we tracked the oxidation of a suitable substrate, such as guaiacol or o-dianisidine. The increase in absorbance resulting from substrate oxidation was quantified at a specific wavelength [24]. Catalase (CAT) activity was determined by monitoring the decomposition of hydrogen peroxide (H_2_O_2_) catalyzed by the enzyme. We quantified the decrease in absorbance at 240 nm resulting from H_2_O_2_ decomposition [25]. For ascorbate peroxidase (APX) activity, we monitored the oxidation of ascorbate in the presence of H_2_O_2_. This was conducted following the method detailed by [26].

### Electrolyte leakage

To perform the analysis, the leaves were initially cleansed with deionized water to eliminate any external impurities. Next, we obtained uniform leaf fragments weighing about one gram each using a steel cylinder with a 1 cm diameter. These leaf fragments were then introduced into individual test tubes, each containing 20 ml of deionized water. The test tubes were kept at a consistent temperature of 25 °C for 24 h, allowing electrolytes to diffuse from the leaf tissues into the water solution. Following the incubation period, we measured the electrical conductivity (EC1) of the water solution using a properly calibrated EC meter. Subsequently, the test tubes were subjected to a 20-min heating process in a water bath set at 120 °C, and the second electrical conductivity (EC2) was recorded [27].

$$\text{Electrolyte Leakage}\;{(\%)}\;\;=\;(\frac{\mathrm{EC1}}{\text{EC2}})\;\times\;100$$

### Leaf and seed K and Ca analysis

Leaf and seed samples were subjected to K (potassium) and Ca (calcium) analysis using a nitric acid and perchloric acid (2:1) digestion method [28]. The digestion process was carried out at 310 °C until the solution become clear like water. Subsequently, the final values for K and Ca were quantified by dilution of samples (100 times) using a Jenway PFP-7 flamephotometer [29].

### Statistical analysis

The data was subjected to standard statistical analysis. To assess the significance of the treatments, a two-way ANOVA was conducted. For treatment comparison, paired comparisons were carried out using the Tukey test at a significance level of *p* ≤ 0.05. In addition, we employed OriginPro software [30] to generate cluster plot convex hulls, hierarchical cluster plots, and calculate Pearson correlations.

## Results

### Growth attributes of plant

**Fig. 1:**
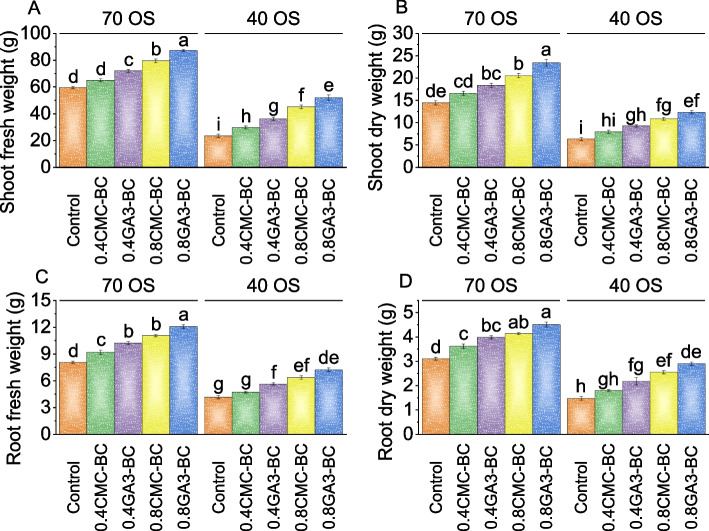
Effect of treatments on shoot fresh weight (**A**), shoot dry weight (**B**), root fresh weight (**C**), and root dry weight (**D**) of cotton cultivated under 70 OS and 40 OS stress. Bars are means of 5 replicates ± SE. Difference letters on bars showed significant changes at *p* ≤ 0.05: Tukey test

In the 70% field capacity condition, applying 0.4 CMC-BC resulted in a 9% increase in shoot fresh weight, while 0.4 GA_3_-BC showed a 21% increase compared to the control. The effects were more pronounced with 0.8 CMC-BC and 0.8 GA_3_-BC, leading to fresh weight increases of 35% and 46%, respectively, parallel to the control under 70% field capacity. Under 40% field capacity, the control group had a fresh weight of 23.31 g. In contrast, 0.4 CMC-BC treatment showed a significant 26.73% increase, and 0.4 GA_3_-BC treatment exhibited a more substantial 54.93% increase. The 0.8 CMC-BC showed a 93.04% increase, and 0.8 GA_3_-BC treatments demonstrated notable increases in shoot fresh weight over the 0.4 CMC-BC, 0.4 GA_3_-BC, and 0.8 CMC-BC treatments and the control under 40% field capacity (Fig. 1A).

In 70% field capacity, the control group exhibited a mean shoot dry weight of 14.37 g. The use of 0.4CMC-BC increased dry weight by 15.17%, while 0.4GA_3_-BC resulted in a substantial 27.63% increase over the control. In the 0.8CMC-BC treatment, dry weight rose by 42.54%, and 0.8GA_3_-BC showed a remarkable 62.68% increase over the control, demonstrating a statistical advantage over 0.4CMC-BC and 0.4GA_3_-BC treatments. In the 40% field capacity group, the control had a shoot dry weight of 6.28 g. The use of 0.4CMC-BC increased dry weight by 25.89%, while 0.4GA_3_-BC led to a substantial 46.84% increase. The application of 0.8CMC-BC showed a 72.36% increase over the control in shoot dry weight (40% field capacity). The most significant increase was observed with 0.8GA_3_-BC, resulting in a remarkable 95.70% increase in shoot dry weight compared to the control and 0.8CMC-BC treatment (Fig. 1B).

In root fresh weight, the 0.8CMC-BC treatment demonstrated a 49.61% increase over the control and 0.4CMC-BC, 0.4GA_3_-BC, and 0.8CMC-BC treatments under 70% field capacity. The 0.4CMC-BC treatment showed a 13.97% increase, 0.4GA_3_-BC resulted in a 26.78% increase, and 0.8CMC-BC treatment exhibited a 37.00% increase compared to the control under 70% field capacity. In the 40% field capacity treatment, 0.4CMC-BC led to a 13.51% increase, while 0.4GA_3_-BC resulted in a more substantial 35.21% increase over the control group with a mean root fresh weight of 4.19 g. The 0.8CMC-BC treatment exhibited a 52.54% rise, and the 0.8GA_3_-BC treatment showed a 73.13% improvement in root fresh weight compared to the control under 40% field capacity-stressed conditions (Fig. 1C).

In 70% field capacity conditions, the control group’s root dry weight was 3.11 g. Contrasting with the control, the 0.4CMC-BC treatment increased root dry weight by 16.60%, while the 0.4GA_3_-BC treatment significantly raised it by 28.37%. In 70% field capacity, the 0.8CMC-BC treatment showed a 33.40% greater root dry weight, and the 0.8GA_3_-BC treatment had the most notable rise at 45.08%. Under 40% field capacity stress, the 0.4CMC-BC treatment resulted in a 22.20% increase in root dry weight, while the 0.4GA_3_-BC treatment showed a 47.31% increase over the control. The 0.8CMC-BC treatment had a 72.20% higher root dry weight in 40% field capacity, and the 0.8GA_3_-BC treatment exhibited a statistically significant 95.74% increase compared to the control (Fig. 1D).

### Chlorophyll and carotenoid content

**Fig. 2:**
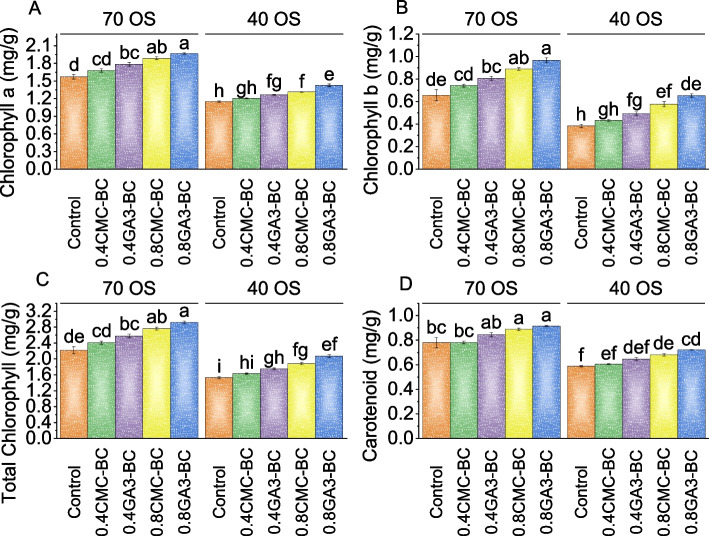
Effect of treatments on chlorophyll a (**A**), chlorophyll b (**B**), total chlorophyll (**C**), and carotenoids (**D**) of cotton cultivated under 70 OS and 40 OS stress. Bars are means of 5 replicates ± SE. Difference letters on bars showed significant changes at *p* ≤ 0.05; Tukey test

In the 70% field capacity treatment group, the chlorophyll a content (mg/g) increased compared to the control (mean 1.57 mg/g): 0.4CMC-BC showed a 6.81% increase, 0.4GA_3_-BC a 13.62% increase, 0.8CMC-BC a 20.21% increase, and 0.8GA_3_-BC the highest increase at 25.32%. In the 40% field capacity condition, the control group had a mean chlorophyll a content of 1.15 mg/g. 0.4CMC-BC exhibited a 4.64% increase, 0.4GA_3_-BC showed a 9.85% increase, 0.8CMC-BC had a 14.20% increase, and 0.8GA_3_-BC had the highest increase in chlorophyll a content at 23.77%, surpassing the control and other treatments (0.4CMC-BC, 0.4GA_3_-BC, and 0.8CMC-BC) under 40% field capacity condition (Fig. 2A).

In the 70% field capacity condition, the control group exhibited an average chlorophyll b content of 0.66 mg/g. At this condition, the 0.4CMC-BC treatment showed a 12.69% increase, 0.4GA_3_-BC demonstrated a 22.33% enhancement, and the 0.8CMC-BC and 0.8GA_3_-BC treatments displayed even more pronounced boosts of 35.03% and 46.70%, respectively, compared to the control. Under osmotic stress (40% field capacity), the 0.4CMC-BC treatment resulted in a 13.04% rise in chlorophyll b content compared to the control. The 0.4GA_3_-BC treatment at 40% field capacity exhibited a substantial 28.70% increase, while 0.8CMC-BC treatment led to a remarkable 50.44% increase, and the 0.8GA_3_-BC treatment showed an impressive 70.44% improvement when compared to the control (Fig. 2B).

In the 70% field capacity condition, the control group’s average total chlorophyll content was 2.22 mg/g. Over the control, 0.4CMC-BC led to an 8.55% increase, while 0.4GA_3_-BC resulted in a 16.19% increase in total chlorophyll content. Meanwhile, 0.8CMC-BC and 0.8GA_3_-BC treatments exhibited even greater increases of 24.59% and 31.63%, respectively, than the control under 70% field capacity. In the 40% field capacity condition, 0.4CMC-BC showed a 6.74% increase, and 0.4GA_3_-BC exhibited a 14.57% increase in total chlorophyll content compared to the control. The 0.8CMC-BC treatment demonstrated a significant 23.26% increase, and the 0.8GA_3_-BC treatment had the highest increase of 35.44% over the 0.4CMC-BC, 0.4GA_3_-BC, and 0.8CMC-BC or control under 40% field capacity-stressed conditions (Fig. 2C).

In 70% field capacity, the control group’s average carotenoid content was 0.78 mg/g. With 0.4CMC-BC treatment, there was a 0.26% increase, and 0.4GA_3_-BC resulted in an 8.28% increase in carotenoid content. The 0.8CMC-BC treatment led to a substantial 13.91% increase, while the 0.8GA_3_-BC treatment exhibited the highest increase of 17.36%. In the 40% field capacity stress condition, the control group showed carotenoid levels of 0.59 mg/g. With 0.4CMC-BC, there was a modest 2.77% increase, 0.4GA_3_-BC led to a more significant 9.77% increase, and 0.8CMC-BC resulted in a 15.71% increase in carotenoid content compared to the 40% field capacity control. The 0.8GA_3_-BC treatment exhibited the highest increase of 22.54% in carotenoids under 40% field capacity compared to the control treatment (Fig. 2D).

### Photosynthetic rate, stomatal conductance, intracellular CO_2_ conc. and transpiration rate

**Fig. 3:**
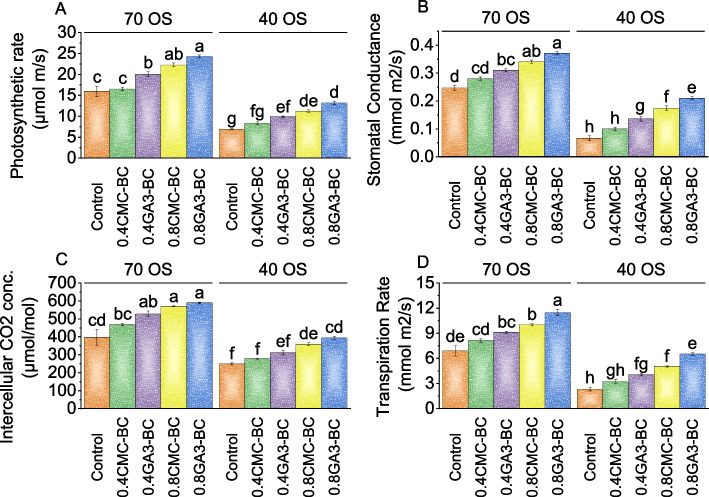
Effect of treatments on photosynthetic rate (**A**), stomatal conductance (**B**), intracellular CO_2_ conc. (**C**), and transpiration rate (**D**) of cotton cultivated under 70 OS and 40 OS stress. Bars are means of 5 replicates ± SE. Difference letters on bars showed significant changes at *p* ≤ 0.05; Tukey test

In 70% field capacity, the control group’s average carotenoid content was 0.78 mg/g. With 0.4CMC-BC treatment, there was a 0.26% increase, and 0.4GA_3_-BC resulted in an 8.28% increase in carotenoid content. The 0.8CMC-BC treatment led to a substantial 13.91% increase, while the 0.8GA_3_-BC treatment exhibited the highest increase of 17.36%. In the 40% field capacity stress condition, the control group showed carotenoid levels of 0.59 mg/g. With 0.4CMC-BC, there was a modest 2.77% increase, 0.4GA_3_-BC led to a more significant 9.77% increase, and 0.8CMC-BC resulted in a 15.71% increase in carotenoid content compared to the 40% field capacity control. The 0.8GA_3_-BC treatment exhibited the highest increase of 22.54% in carotenoids under 40% field capacity compared to the control treatment (Fig. 3A).

In the presence of 0.4CMC-BC, there was a 13.51% increase in stomatal conductance at 70% field capacity, which further improved to 25.67% when 0.4GA_3_-BC was applied over the control. The 0.8CMC-BC treatment resulted in a substantial 37.84% increase, and the application of 0.8GA_3_-BC yielded the most significant improvement, in comparison to the control group, with a 50.00% increase in stomatal conductance at 70% field capacity. Under 40% field capacity, stomatal conductance increased by 49.99% with 0.4CMC-BC, 104.99% with 0.4GA_3_-BC, 159.98% with 0.8CMC-BC, and 214.98% with 0.8GA_3_-BC contrasted to the control (Fig. 3B).

Under 70% field capacity conditions, the control exhibited a mean intercellular CO2 concentration of 398.47 µmol/mol. The addition of 0.4CMC-BC treatment showed a 17.96% increase, while the 0.4GA_3_-BC treatment demonstrated a more substantial 32.65% rise. The 0.8CMC-BC treatment led to a remarkable 43.37% increase in intercellular CO2 concentration, and the 0.8GA_3_-BC treatment resulted in the highest increase of 48.25% over the control under 70% field capacity. Similarly, at 40% field capacity, the 0.4CMC-BC treatment showed an 11.35% increase over the control, and the 0.4GA_3_-BC treatment exhibited a 24.74% rise in intercellular CO2 concentration. The 0.8CMC-BC treatment demonstrated a substantial 43.69% increase, and the 0.8GA_3_-BC treatment led to the most significant change with a 57.99% increase in intercellular CO2 concentration related to the control group under 40% field capacity (Fig. 3C).

In the 70% field capacity condition, the control had a mean transpiration rate of 6.92 mmol m2/s. Treatment 0.4CMC-BC showed a 17.97% increase compared to the control, while the 0.4GA_3_-BC treatment exhibited a 32.08% increase at 70% field capacity. The 0.8CMC-BC treatment resulted in a substantial 45.42% increase in transpiration rate, and the 0.8GA_3_-BC treatment showed the highest percentage increase, with a remarkable 66.18% rise compared to the control (70% field capacity). For the 40% field capacity condition, the 0.4CMC-BC treatment resulted in a substantial 37.71% increase in transpiration rate compared to the control. The 0.4GA_3_-BC treatment showed an impressive 71.93% increase, and the 0.8CMC-BC treatment exhibited a remarkable 112.43% increase. The 0.8GA_3_-BC treatment displayed the highest percentage increase of 174.44% when compared to the control under 40% field capacity (Fig. 3D).

### Total protein, electrolyte leakage, total phenolics and flavonoids

**Fig. 4:**
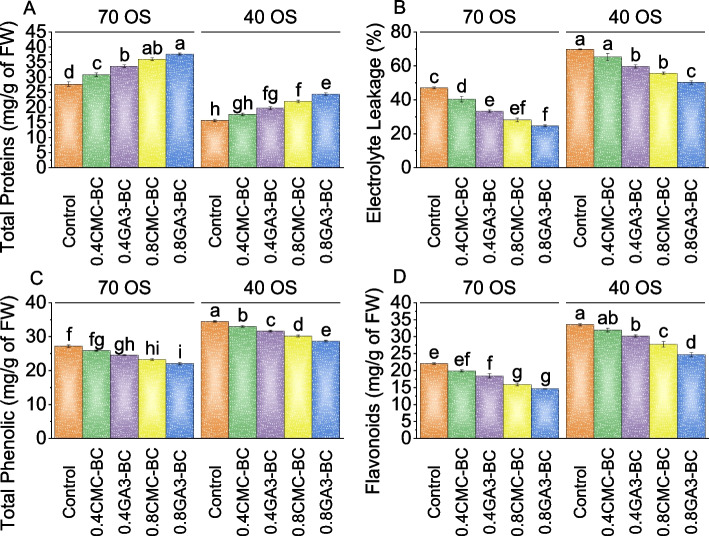
Effect of treatments on total protein (**A**), electrolyte leakage (**B**), total phenolics (**C**), and flavonoids (**D**) of cotton cultivated under 70 OS and 40 OS stress. Bars are means of 5 replicates ± SE. Difference letters on bars showed significant changes at *p* ≤ 0.05; Tukey test

For the 70% field capacity group, the control had a mean total protein level of 27.63 mg/g of FW. The 0.4CMC-BC treatment showed an 11.18% increase, while the 0.4GA_3_-BC treatment exhibited a 21.50% increase compared to the control under 70% field capacity. The 0.8CMC-BC treatment resulted in a substantial 29.88% increase compared to the 70% field capacity control, and the 0.8GA_3_-BC treatment showed the highest increase of 36.01%. In the 40% field capacity group, the control had a baseline total protein level of 15.65 mg/g of FW. The 0.4CMC-BC treatment led to a 12.84% increase in total proteins, while the 0.4GA_3_-BC treatment resulted in a significant 26.19% increase. The 0.8CMC-BC treatment exhibited a substantial 40.12% rise over the 40% field capacity control, and the 0.8GA_3_-BC treatment showed the highest increase of 55.73% (Fig. 4A).

In contrast to the control group, electrolyte leakage was 47.03% at the 70% field capacity stress level. At 70% field capacity, the 0.4CMC-BC treatment resulted in a 16.45% decrease in electrolyte leakage, and the 0.4GA_3_-BC treatment exhibited a more pronounced 41.12% reduction compared to the control. Over the control, the 0.8CMC-BC treatment showed even greater effectiveness, with a substantial 67.14% decrease in electrolyte leakage, and the 0.8GA_3_-BC treatment had the most significant effect, reducing electrolyte leakage by a remarkable 90.60%. At the 40% field capacity stress level, the control group had an initial electrolyte leakage percentage of 69.63%. In comparison to the control, the 0.4CMC-BC treatment led to a 7.00% decrease in leakage, the 0.4GA_3_-BC treatment resulted in a 17.00% reduction, the 0.8CMC-BC treatment showed a considerable 25.35% decrease, while the 0.8GA_3_-BC treatment was the most effective, with a notable 39.03% reduction in electrolyte leakage under 40% field capacity stress (Fig. 4B).

Under the 70% field capacity condition, the control group had a total phenolic content of 27.26 mg/g of FW. With the 0.4CMC-BC treatment, the total phenolic content decreased by 5.06% compared to the 70% field capacity control. The 0.4GA_3_-BC treatment resulted in a 10.72% decrease, while the 0.8CMC-BC and 0.8GA_3_-BC treatments led to more substantial decreases of 16.78% and 23.31%, respectively, compared to the control (70% field capacity). For the 40% field capacity stress condition, the control group displayed a total phenolic content of 34.53 mg/g of FW. Under the influence of the 0.4CMC-BC treatment, there was a 4.39% reduction in total phenolic content compared to the control, and the 0.4GA_3_-BC treatment yielded an 8.93% decrease. The 0.8CMC-BC and 0.8GA_3_-BC treatments resulted in larger declines of 14.15% and 20.30%, respectively, when contrasted with the control group (Fig. 4C).

Flavonoid levels in the 70% field capacity control group showed an average of 22.15 mg/g of FW. In contrast, the 0.4GA_3-_BC treatment exhibited a substantial 19.64% decrease in flavonoid content compared to the baseline treatment. The 0.8CMC-BC treatment resulted in a significant 38.55% reduction, while the 0.8GA_3_-BC treatment showed an even greater decrease of 50.61% in flavonoid concentration compared to the 70% field capacity control. For the 40% field capacity group, the control exhibited a mean flavonoid content of 33.61 mg/g of FW, and the addition of 0.4CMC-BC treatment showed a 5.03% drop in flavonoid content. The 0.4GA_3_-BC treatment saw an 11.10% decrease, while the 0.8CMC-BC treatment displayed a more substantial 20.83% reduction than the 40% field capacity control. The 0.8GA_3_-BC treatment recorded the most significant decrease in flavonoid content with a 35.89% reduction compared to the control group (Fig. 4D).

### Antioxidants

**Fig. 5:**
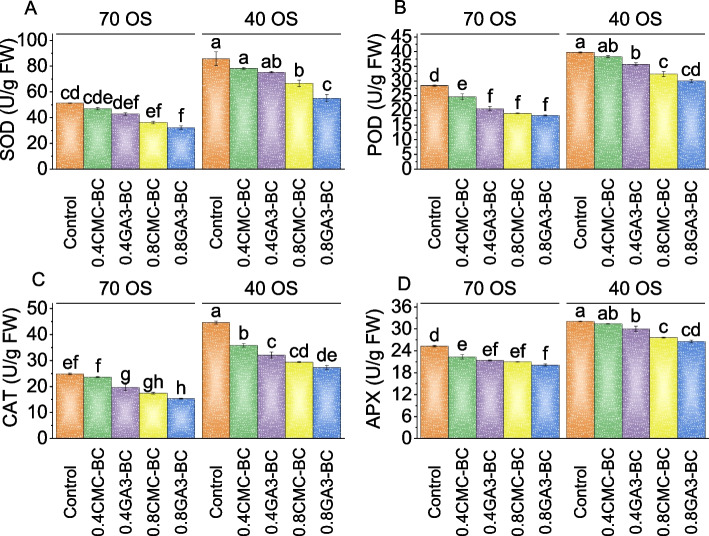
Effect of treatments on superoxide dismutase (SOD) (**A**), peroxidase (POD) (**B**), catalase (CAT) (**C**), and APX (**D**) of cotton cultivated under 70 OS and 40 OS stress. Bars are means of 5 replicates ± SE. Difference letters on bars showed significant changes at *p* ≤ 0.05; Tukey test

The control had a mean Superoxide Dismutase (SOD) activity of 51.30 U/g FW. At the 70% Osmotic Stress (OS) level, compared to the control, treatment with 0.4CMC-BC exhibited a 9.26% decrease in SOD activity, while 0.4GA_3_-BC resulted in a more substantial 20.02% decrease. The 0.8CMC-BC treatment showed a considerable 41.86% reduction above the 70% field capacity control, and the 0.8GA_3_-BC treatment resulted in the most significant decrease at 59.42%. At the 40% field capacity level, 0.4CMC-BC exhibited a 9.48% reduction in SOD activity, and 0.4GA_3_-BC resulted in a 13.88% decrease. The 0.8CMC-BC treatment showed a substantial 28.58% decrease from the control in 40% field capacity-stressed conditions, and the 0.8GA_3_-BC treatment had the most prominent decrease of 55.32% (Fig. 5A).

Peroxidase (POD) results displayed significant variations in response to different treatments. At 70% field capacity, the control group exhibited a POD activity of 28.36 U/g FW. In comparison to the control, the treatment with 0.4CMC-BC resulted in a 15.28% decrease in POD activity, while 0.4GA_3_-BC and 0.8CMC-BC treatments caused more substantial reductions of 37.98% and 49.15%, respectively. The 0.8GA_3_-BC treatment exhibited the highest decrease at 55.48% over the 70% field capacity control. At 40% field capacity, the control group had a POD activity of 39.65 U/g FW. Treatment with 0.4CMC-BC resulted in a 3.79% decrease, 0.4GA_3_-BC caused an 11.32% reduction compared to the control, and the 0.8CMC-BC treatment led to a 22.90% decrease in POD activity. The most significant decrease in POD activity was observed in the 0.8GA_3_-BC treatment, with a 32.60% reduction compared to the control in 40% field capacity stress (Fig. 5B).

In the 70% field capacity treatment group, catalase (CAT) activity showed a significant decrease when treated with 0.4CMC-BC by 5.02%, and a substantial decrease of 26.40% with 0.4GA_3_-BC, while 0.8CMC-BC and 0.8GA_3_-BC treatments resulted in even more substantial reductions of 42.53% and 61.76%, respectively, related to the control. Similarly, in the 40% FIELD CAPACITY treatment group, CAT activity was notably reduced by 24.43% with 0.4CMC-BC and 38.97% with 0.4GA_3_-BC, while 0.8CMC-BC and 0.8GA_3_-BC treatments led to substantial decreases of 51.56% and 63.33%, respectively, contrasted to the control (Fig. 5C).

For 70% field capacity, the use of 0.4CMC-BC led to a 13.10% decrease, while 0.4GA_3_-BC caused an 18.25% decrease in APX activity compared to the control. The 0.8CMC-BC treatment exhibited the highest decrease from the control in 70% field capacity, with a 20.44% reduction, and the 0.8GA_3_-BC treatment resulted in the most significant decrease at 25.68%. Similarly, at 40% field capacity, the trends persisted, with 0.4CMC-BC leading to a 2.12% decrease, 0.4GA_3_-BC causing a 6.85% decrease, 0.8CMC-BC resulting in a 15.96% decrease, and 0.8GA_3_-BC leading to the highest decrease of 20.67% in APX activity compared to the control (Fig. 5D).

### Leaf and seed (K and Ca)

**Fig. 6:**
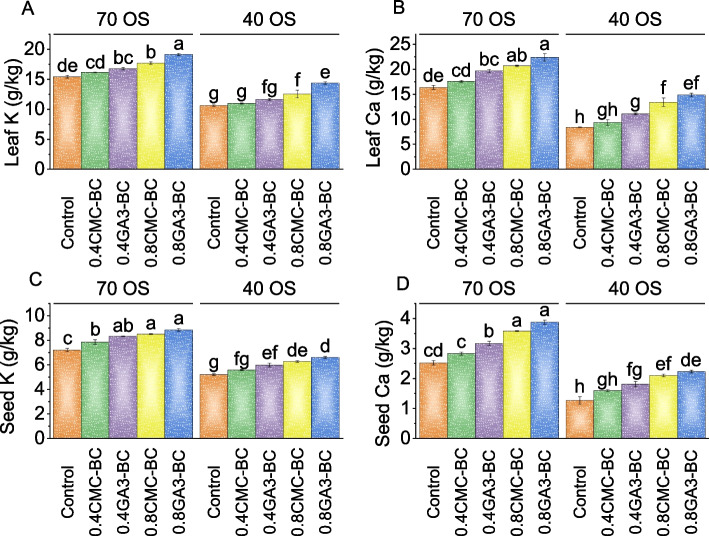
Effect of treatments on leaf K (**A**), leaf Ca (**B**), seed K (**C**), and seed Ca (**D**) of cotton cultivated under 70 OS and 40 OS stress. Bars are means of 5 replicates ± SE. Difference letters on bars showed significant changes at *p* ≤ 0.05; Tukey test

Leaf K content in the 70% field capacity control group was 15.37 g/kg, while treatment with 0.4CMC-BC resulted in a 4.79% increase over the control, and 0.4GA_3_-BC treatment showed an 8.70% increase in leaf K content. The 0.8CMC-BC treatment exhibited the most significant increase, with a 14.86% rise in leaf K content, and the 0.8GA_3_-BC treatment showed a substantial 24.26% increase compared to the 70% field capacity control. In the 40% field capacity group, the control had a leaf K content of 10.61 g/kg, with a 3.31% increase when treated with 0.4CMC-BC and a 9.54% increase with 0.4GA_3_-BC. The 0.8CMC-BC treatment resulted in an 18.10% increase, and the 0.8GA_3_-BC treatment showed a notable 34.97% increase in leaf K content compared to the control (Fig. 6A).

In 70% field capacity over the control, the 0.4CMC-BC treatment improved calcium content by 7.46%, while the 0.4GA_3_-BC treatment significantly raised calcium levels by 20.01%. The 0.8CMC-BC treatment resulted in a remarkable 26.19% increase in leaf calcium content, and the most substantial effect was observed in the 0.8GA_3_-BC treatment, which showed a remarkable 36.70% increase compared to the control in 70% field capacity. In the 40% field capacity, the control group had a baseline calcium content of 8.40 g/kg. The 0.4CMC-BC treatment demonstrated an 11.33% increase, and the 0.4GA_3_-BC treatment exhibited a notable 31.87% increase in leaf calcium levels over the 40% field capacity control. In comparison, the 0.8CMC-BC treatment showed a substantial 59.77% increase, raising calcium content to 13.42 g/kg. Finally, the 0.8GA_3_-BC treatment yielded the most significant impact, with an impressive 76.86% increase in leaf calcium content compared to the baseline treatment in 40% field capacity (Fig. 6B).

At 70% field capacity, the control group had an average seed K content of 7.23 g/kg. The use of 0.4CMC-BC resulted in an 8.82% increase in seed K content, while 0.4GA_3_-BC caused a substantial 15.46% increase over the control group in 70% field capacity. A higher stress level of 0.8CMC-BC led to an 18.03% increase, and 0.8GA_3_-BC showed the most significant improvement with a 22.70% increase compared to the control. When the stress level was reduced to 40% field capacity, 0.4CMC-BC showed a 7.32% increase, 0.4GA_3_-BC exhibited a 14.90% increase, and 0.8CMC-BC resulted in a 20.56% increase in seed K content parallel to the control under 40% field capacity stress. The highest increase was seen with 0.8GA_3_-BC at 26.92% compared to the 40% field capacity control (Fig. 6C).

In comparison to the control, treatment with 0.4CMC-BC raised the seed Ca content in the 70% field capacity by 12.15%, and with 0.4GA_3_-BC, it rose by 25.61%. The Ca content exhibited a more substantial increase of 41.77% with 0.8CMC-BC and 53.54% with 0.8GA_3_-BC compared to the control. Under 40% field capacity stress treatment, the Ca content increased by 25.53% with 0.4CMC-BC and 42.50% with 0.4GA_3_-BC. The most significant increase was observed with 0.8CMC-BC, where the Ca content increased by 65.21%, and 0.8GA_3_-BC treatment resulted in a 75.38% increase in Ca content compared to the 40% field capacity control (Fig. 6D).

### Convex hull and hierarchical cluster analysis

**Fig. 7:**
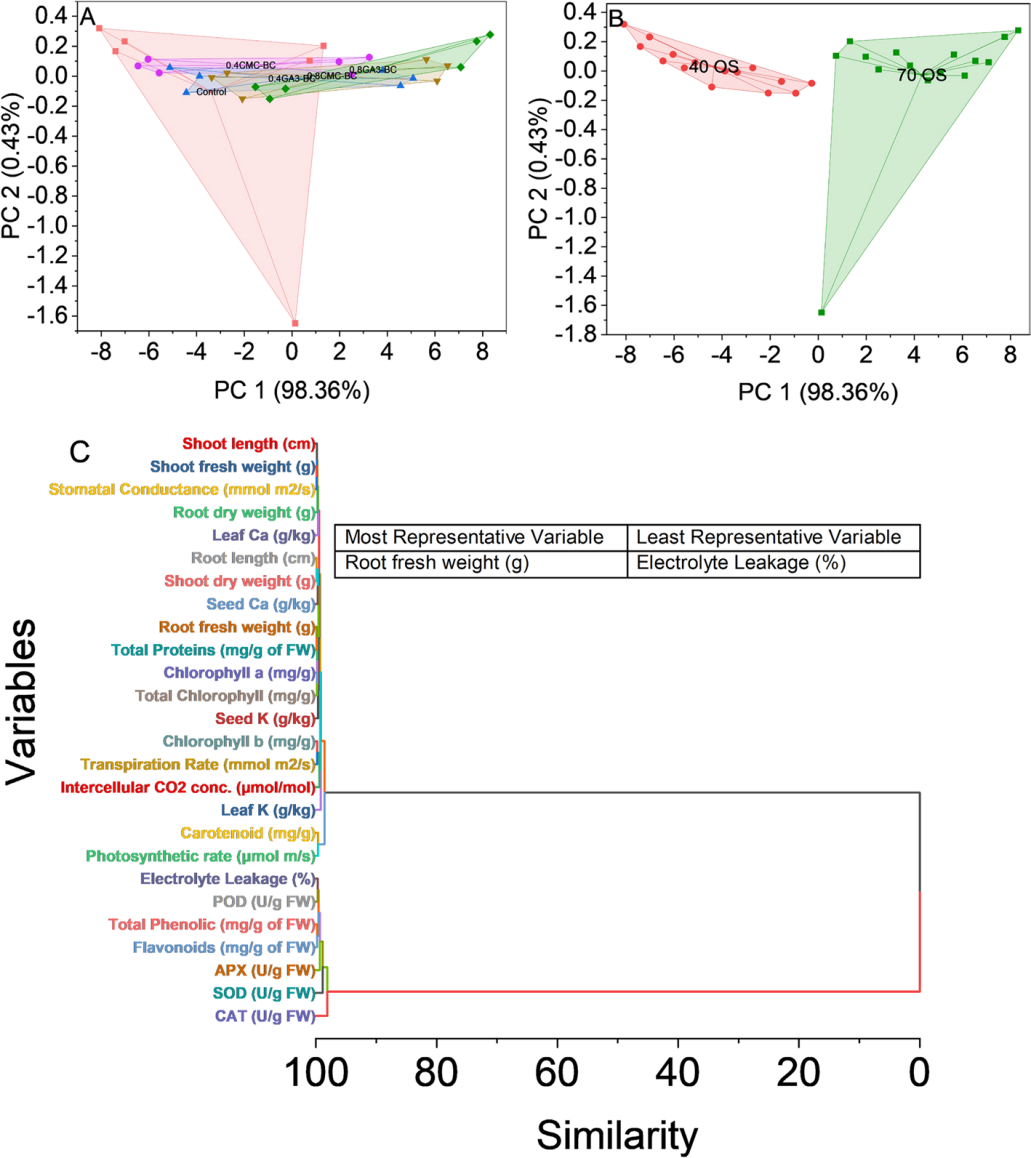
Cluster plot convex hull for treatments (**A**), osmotic stress levels (**B**), and hierarchical cluster plot (**C**) for studied attributes

The study, employing convex hull analysis, unveils insights into how different treatments impact data distribution in the principal component (PC) space. The control group shows a tightly clustered distribution in PC space, mainly influenced by PC 1 (98.36%), reflecting a consistent response. Limited PC 2 influence (0.43%) suggests treatment effects primarily along the PC 1 axis. Treated groups (0.4CMC-BC, 0.4GA_3_-BC, 0.8CMC-BC, 0.8GA_3_-BC) display scattered distributions, signifying diverse effects and induced variations in responses, leading to a broader dispersion of data points. The separation of treated groups underscores specific treatment influences on measured parameters. Variations in PC 1 and PC 2 scores within treated groups, notably in 0.4CMC-BC, provide insights into treatment-specific effects on system responses (Fig. 7A).

The convex hull analysis of the dataset unveils distinct patterns in data point distribution in the two principal components (PC 1 and PC 2) space, particularly under the stress conditions of 70% field capacity and 40% field capacity. In the 70% field capacity stress condition, data points formed a compact cluster with PC 1 explaining approximately 98.36% of the variance, suggesting a consistent and uniform response. This alignment with the stress condition implies minimal variance in treatment response. Conversely, the 40% field capacity stress condition exhibited a scattered cluster of data points, indicating a diverse range of responses. While PC 1 accounted for a significant proportion of variance (98.36%), the data points under 40% field capacity stress showed a broader range of responses compared to the 70% field capacity condition, emphasizing the varied impact of the stress conditions on the system (Fig. 7B).

The hierarchical cluster analysis of the dataset identifies distinct patterns in variable relationships, revealing potential interdependencies. The dendrogram groups variables based on similar response patterns, indicating key associations. Shoot Fresh Weight and Stomatal Conductance cluster together, as do root length, shoot dry weight, and root fresh weight. Total proteins and shoot length share a cluster, as do chlorophyll a and Total Chlorophyll, total phenolic and flavonoids, and Seed Ca with Seed K. Leaf Ca and Electrolyte Leakage are paired, and Carotenoid and photosynthetic rates exhibit similar responses. Intercellular CO2 Concentration and APX show correlated patterns, as do Leaf K and SOD. CAT stands out in its unique response cluster. These insights offer valuable guidance for further exploration into the mechanisms and biological significance of these variable interactions (Fig. 7C).

### Pearson correlation analysis

**Fig. 8:**
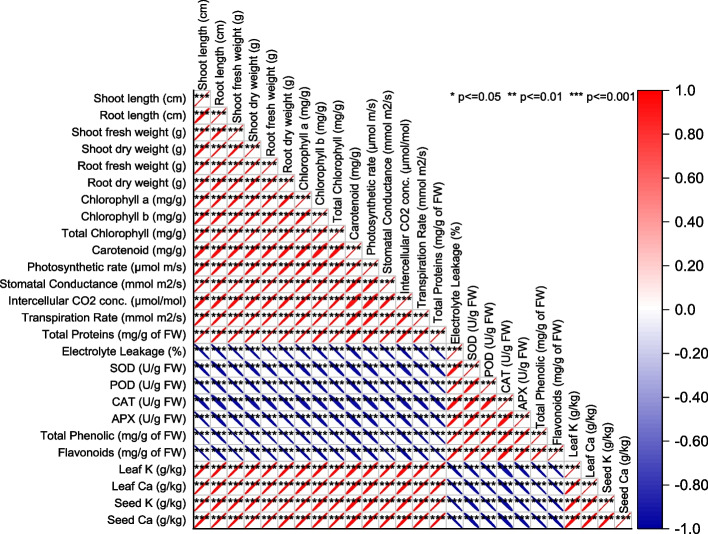
Pearson correlation for studied attributes

The Pearson correlation analysis reveals insightful relationships between variables. Strong positive correlations, such as those between shoot fresh weight and shoot length, root length, and other shoot and root-related traits, indicate coordinated responses within these groups. Conversely, strong negative correlations, like electrolyte leakage with total proteins and total phenolic, suggest inverse relationships and potential trade-offs in the system. Weaker correlations, like the positive association between Leaf K and Seed Ca, provide a nuanced perspective. The emergence of variable clusters with strong positive correlations underscores shared influencing factors or biological mechanisms, particularly seen in groupings of shoot and root characteristics, as well as chlorophyll content, indicating parallel responses to treatments or conditions (Fig. 8).

## Discussion

The current study was conducted to evaluate the combine effect of Gibberellic acid + Biochar and CMC + Biochar on the cotton production under no drought (70%) and drought stress (50%) conditions in greenhouse experiment.

The root fresh weight reflects the plant’s ability to develop roots, essential for nutrient uptake, water absorption, and overall plant health (Fig. 1). Osmotic stress significantly impacted root growth and water balance in plants, making root fresh weight a valuable tool for evaluating the effectiveness of treatments in mitigating its adverse effects [31]. The study found that gibberellic acid (GA_3_) significantly impacted root growth, with the 0.8GA_3_-BC application showing the most substantial increase in both root fresh and dry weights (Fig. 1). Increased root biomass enhanced the plant’s ability to access water and nutrients, even under osmotic stress conditions [32]. Furthermore, the treatments applied in our study appeared to mitigate the negative impact of osmotic stress on chlorophyll and carotenoid content in cotton plants (Fig. 2). These treatments likely achieved this through mechanisms such as protecting chlorophyll molecules, promoting pigment synthesis, and enhancing antioxidant defenses [33]. Chlorophyll, a, crucial for photosynthesis, observed an increase due to treatments that mitigated osmotic stress. They enhanced water uptake and protected chlorophyll molecules. The carotenoids, important for protecting plants from oxidative stress and aiding in photosynthesis, saw increased content in treated plants, indicating enhanced defense mechanisms against osmotic stress [34]. The findings revealed that the 0.8GA_3_-BC significantly increased carotenoid content in conditions of 70 osmotic stress, suggesting its role in activating antioxidant pathways [35]. The 0.4CMC-BC, 0.4GA_3_-BC, 0.8CMC-BC, and 0.8GA_3_-BC, positively influenced plant growth parameters such as photosynthetic rate, stomatal conductance, intracellular CO_2_ concentration, and transpiration rate (Fig. 3). The enhanced photosynthetic rate was attributed to increased chlorophyll content, improved stomatal conductance, and enhanced carbon dioxide utilization [36]. Furthermore, these treatments appeared to improve stomatal regulation, water use efficiency, and plant growth (Fig. 3). This might be potentially due to changes in guard cell turgor pressure influenced by the plant’s osmotic potential. The study also observed an increase in total protein levels under these treatments, indicating a positive impact on plant protein synthesis, likely due to improved nutrient uptake (Fig. 4). This enhancement in protein synthesis contributed to overall plant growth. In terms of cellular health, reduced electrolyte leakage was noted, implying improved membrane integrity, and reduced cellular damage under osmotic stress [37]. Such improvements are common responses in plants under stress conditions, potentially a result of treatments reducing oxidative stress and membrane damage through their antioxidant properties. Interestingly, while total phenolic and flavonoid content decreased in plants under these treatments (Fig. 4), it might be a possible shift in secondary metabolism, with these metabolites typically produced in response to stress. This reduction might be attributed to reduced stress levels or changes in resource allocation within the plant. The decrease in antioxidant activity, it can be considered a positive sign, indicating reduced oxidative stress and improved plant stress management. The treatments might reduce the generation of reactive oxygen species (ROS) or enhance the plant’s natural antioxidant defense mechanisms [38]. Moreover, the treatments led to an increase in leaf potassium and calcium content (Fig. 6), suggesting improvements in nutrient uptake and translocation. Potassium is crucial for various physiological processes like water uptake and enzyme activation, while calcium is involved in maintaining cell wall stability and signaling. This indicates that these treatments might be enhanced root function and nutrient transport within the plant which resulted in more uptake of nutrients. The practical significance of these findings is significant. Farmers facing stress-inducing conditions may find value in utilizing 0.8GA_3_-BC as a focused remedy to boost crop performance and reduce yield losses. Adopting this treatment has the potential to result in higher crop yields and enhanced overall farm productivity, especially in areas where osmotic stress consistently hinders cotton cultivation.

## Conclusion

In conclusion, the application of 0.8GA3-BC emerges as a promising strategy for mitigating osmotic stress in cotton cultivation, showcasing its efficacy as a potent soil amendment. The treatment not only yielded significant enhancements in critical parameters such as shoot and root weights, chlorophyll levels, photosynthetic and transpiration rates, but also demonstrated its potential to elevate internal CO2 concentration in cotton plants under stress conditions. These compelling findings underscore the capacity of the 0.8GA3-BC treatment to bolster agricultural resilience and productivity in environments prone to stress. The practical implications of these results are noteworthy. Farmers grappling with stress-prone conditions can potentially leverage 0.8GA3-BC as a targeted solution to enhance crop performance and mitigate yield losses. Implementing this treatment could translate into increased crop output and improved overall farm productivity, particularly in regions where osmotic stress poses a persistent challenge to cotton cultivation.

## Data Availability

All data generated or analysed during this study are included in this published article.

## References

[1] Pakzad R, Fatehi F, Kalantar M, Maleki M (2023). Proteomics approach to investigating osmotic stress effects on pistachio. Front Plant Sci.

[2] Konieczna W, Mierek-Adamska A, Warchoł M, Skrzypek E, Dąbrowska GB (2023). The involvement of metallothioneins and stress markers in response to osmotic stress in Avena sativa L. J Agron Crop Sci.

[3] Alharbi K, Hafez EM, Omara AE-D, Osman HS (2023). Mitigating osmotic stress and enhancing developmental productivity processes in cotton through integrative use of vermicompost and cyanobacteria. Plants..

[4] Akalin GO, Pulat M (2020). Controlled release behavior of zinc-loaded carboxymethyl cellulose and carrageenan hydrogels and their effects on wheatgrass growth. J Polym Res.

[5] Wang Y, Gao M, Chen H, Chen Y, Wang L, Wang R (2023). Fertigation and carboxymethyl cellulose applications enhance water-use efficiency, improving soil available nutrients and maize yield in salt-affected soil. Sustainability.

[6] Alharby HF, Rizwan M, Iftikhar A, Hussaini KM, urRehman MZ, Bamagoos AA (2021). Effect of gibberellic acid and titanium dioxide nanoparticles on growth, antioxidant defense system and mineral nutrient uptake in wheat. Ecotoxicol Environ Saf..

[7] Iftikhar A, Rizwan M, Adrees M, Ali S, urRehman MZ, Qayyum MF (2020). Effect of gibberellic acid on growth, biomass, and antioxidant defense system of wheat (Triticum aestivum L.) under cerium oxide nanoparticle stress. Environ Sci Pollut Res.

[8] AL-Huqail AA, Alshehri D, Nawaz R, Irshad MA, Iftikhar A, Hussaini KM (2023). The effect of gibberellic acid on wheat growth and nutrient uptake under combined stress of cerium, zinc and titanium dioxide nanoparticles. Chemosphere.

[9] Bhat JA, Basit F, Alyemeni MN, Mansoor S, Kaya C, Ahmad P (2023). Gibberellic acid mitigates nickel stress in soybean by cell wall fixation and regulating oxidative stress metabolism and glyoxalase system. Plant Physiol Biochem.

[10] Khan MN, Khan Z, Luo T, Liu J, Rizwan M, Zhang J (2020). Seed priming with gibberellic acid and melatonin in rapeseed: Consequences for improving yield and seed quality under drought and non-stress conditions. Ind Crops Prod.

[11] Hasan S, Sehar Z, Khan NA (2020). Gibberellic acid and sulfur-mediated reversal of cadmium-inhibited photosynthetic performance in Mungbean (Vigna radiata L.) involves nitric oxide. J Plant Growth Regul.

[12] Dai Y, Zheng H, Jiang Z, Xing B (2020). Combined effects of biochar properties and soil conditions on plant growth: a meta-analysis. Sci Total Environ.

[13] Purakayastha TJ, Bera T, Bhaduri D, Sarkar B, Mandal S, Wade P (2019). A review on biochar modulated soil condition improvements and nutrient dynamics concerning crop yields: pathways to climate change mitigation and global food security. Chemosphere.

[14] Mclaughlin H. Characterizing biochars prior to addition to soils version I. 2010;2010:1–8.

[15] Rhoades JD. Salinity: electrical conductivity and total dissolved solids. In: Sparks DL, Page AL, Helmke PA, Loeppert RH, Soltanpour PN, Tabatabai MA, et al., editors. Methods of Soil Analysis, Part 3, Chemical Methods. Madison: Soil Science Society of America; 1996. p. 417–35.

[16] Schouwenburg JC, Walinga I. Methods of analysis of plant material. 1978.

[17] Miller RS, Harstad K, Bellan J (1998). Evaluation of equilibrium and non-equilibrium evaporation models for many-droplet gas-liquid flow simulations. Int J Multiph Flow.

[18] Pratt PF, Norman AG (2016). Potassium. Methods of soil analysis, part 2: chemical and microbiological properties.

[19] Ahmad I, Akhtar MJ, Zahir ZA, Naveed M, Mitter B, Sessitsch A (2014). Cadmium-tolerant bacteria induce metal stress tolerance in cereals. Environ Sci Pollut Res.

[20] Boutraa T, Akhkha A, Al-Shoaibi AA, Alhejeli AM (2010). Effect of water stress on growth and water use efficiency (WUE) of some wheat cultivars (Triticum durum) grown in Saudi Arabia. J Taibah Univ Sci.

[21] Arnon DI (1949). Copper enzymes in isolated chloroplasts. Polyphenoloxidase in Beta vulgaris. Plant Physiol.

[22] Nazar R, Khan MIR, Iqbal N, Masood A, Khan NA (2014). Involvement of ethylene in reversal of salt-inhibited photosynthesis by sulfur in mustard. Physiol Plant.

[23] Dhindsa RS, Plumb-Dhindsa PL, Reid DM (1982). Leaf senescence and lipid peroxidation: effects of some phytohormones, and scavengers of free radicals and singlet oxygen. Physiol Plant.

[24] Hori M, Kondo H, Ariyoshi N, Yamada H, Hiratsuka A, Watabe T (1997). Changes in the hepatic glutathione peroxidase redox system produced by coplanar polychlorinated biphenyls in Ah-responsive and-less-responsive strains of mice: mechanism and implications for toxicity. Environ Toxicol Pharmacol.

[25] Aebi H (1984). Catalase in vitro. Meth. Enzymol..

[26] Nakano Y, Asada K (1981). Hydrogen peroxide is scavenged by ascorbate-specific peroxidase in spinach chloroplasts. Plant Cell Physiol.

[27] Lutts S, Kinet JM, Bouharmont J (1996). NaCl-induced senescence in leaves of rice (Oryza sativaL.) cultivars differing in salinity resistance. Ann Bot.

[28] Miller R, Kalra Y (1997). Nitric-perchloric acid wet digestion in an open vessel. Handbook of reference methods for plant analysis.

[29] Donald AH, Hanson D, Kalra Y (1998). Determination of potassium and sodium by flame emmision spectrophotometery. Handbook of reference methods for plant analysis.

[30] OriginLab Corporation (2021). OriginPro.

[31] Ashraf MA, Rasheed R, Rizwan M, Hussain I, Aslam R, Qureshi FF (2023). Effect of exogenous taurine on pea (Pisum sativum L.) plants under salinity and iron deficiency stress. Environ Res.

[32] Li S, Wang X, Liu X, Thompson AJ, Liu F (2022). Elevated CO2 and high endogenous ABA level alleviate PEG-induced short-term osmotic stress in tomato plants. Environ Exp Bot.

[33] Abeed AHA, AL-Huqail AA, Albalawi S, Alghamdi SA, Ali B, Alghanem SMS (2023). Calcium nanoparticles mitigate severe salt stress in Solanum lycopersicon by instigating the antioxidant defense system and renovating the protein profile. South Afr J Bot.

[34] Ahmad R, Alsahli AA, Alansi S, Altaf MA (2023). Exogenous melatonin confers drought stress by promoting plant growth, photosynthetic efficiency and antioxidant defense system of pea (Pisum sativum L.). Sci Hortic (Amsterdam).

[35] Dixit M, Pandit A (2023). Role of antioxidants in the prevention of cancer: a comprehensive review.

[36] Lupitu A, Moisa C, Gavrilaş S, Dochia M, Chambre D, Ciutină V (2022). The influence of elevated CO2 on volatile emissions, photosynthetic characteristics, and pigment content in Brassicaceae plants species and varieties. Plants.

[37] Ali S, Gill RA, Ulhassan Z, Zhang N, Hussain S, Zhang K (2023). Exogenously applied melatonin enhanced the tolerance of Brassica napus against cobalt toxicity by modulating antioxidant defense, osmotic adjustment, and expression of stress response genes. Ecotoxicol Environ Saf.

[38] Tan Y, Duan Y, Chi Q, Wang R, Yin Y, Cui D (2023). The role of reactive oxygen species in plant response to radiation. Int J Mol Sci.

